# Smooth muscle-derived macrophage-like cells contribute to multiple cell lineages in the atherosclerotic plaque

**DOI:** 10.1038/s41421-021-00328-4

**Published:** 2021-11-23

**Authors:** Yi Li, Huan Zhu, Qianyu Zhang, Ximeng Han, Zhenqian Zhang, Linghong Shen, Lixin Wang, Kathy O. Lui, Ben He, Bin Zhou

**Affiliations:** 1 Department of Cardiology, Shanghai Chest Hospital, Shanghai Jiaotong University, Shanghai, China; 2 State Key Laboratory of Cell Biology, Shanghai Institute of Biochemistry and Cell Biology, Center for Excellence in Molecular Cell Science, Chinese Academy of Sciences, University of Chinese Academy of Sciences, Shanghai, China; 3 School of Life Science and Technology, ShanghaiTech University, 100 Haike Road, Shanghai, China; 4 Department of Cardiac Surgery, Zhongshan Hospital, Fudan University, Shanghai, China; 5 Department of Chemical Pathology; and Li Ka Shing Institute of Health Sciences, The Chinese University of Hong Kong, Prince of Wales Hospital, Shatin, Hong Kong SAR, China; 6 School of Life Science, Hangzhou Institute for Advanced Study, University of Chinese Academy of Sciences, Hangzhou, Zhejiang, China

**Keywords:** Stem-cell differentiation, Mesenchymal stem cells

Dear Editor,

Atherosclerotic plaques are formed by lipid-rich molecules on the arterial wall that narrow the arteries over time, leading to myocardial infarction and stroke. Unraveling the origin and plasticity of various cell types that critically participate in all phases of plaque formation and destabilization is essential in understanding the pathogenesis of atherosclerosis. Smooth muscle cells (SMCs) in the plaques are derived from preexisting SMCs in the medial layer of vessel, and a subset of these cells in the advanced atherosclerotic plaques can differentiate into macrophage-like cells or foam cells^1–3^. Genetic lineage tracing studies demonstrate that some descendants of SMCs may no longer express SMC markers and convert to macrophage-like cells^2,3^. However, whether these SMC-derived macrophage-like cells adopt a stable or transient macrophage cell fate during the progression of atherosclerosis remains controversial and unclear^4^. Here, we developed a dual genetic lineage approach to specifically trace lineage conversion of SMC-derived macrophage-like cells, if any, and found that a subset of these cells re-adopted the SMC lineage in the fibrous cap, contributing to fibroblasts and pericytes in the plaque.

To specifically trace SMC-derived macrophage-like cells, we used dual orthogonal recombination systems of Dre-rox and Cre-loxP as previously reported^5^. We first generated a *Myh11-Dre* knock-in mouse line by homologous recombination using CRISPR/Cas9 and crossed it with the rox reporter *R26-rox-ZsGreen* line (Supplementary Fig. S1a, b). Immunostaining for ZsGreen and SMC markers such as SMA, SM22, CNN1, and smMHC revealed that *Myh11-Dre* efficiently and specifically targeted aortic SMCs (Supplementary Fig. S1c, d). We next generated a *CD11b-CrexER* knock-in mouse line by targeting Cre-rox-ER-rox (CrexER) into the CD11b gene locus (Supplementary Fig. S1e) and crossed it with the loxP reporter *R26-loxP-tdTomato* line (*R26-tdT*, Supplementary Fig. S1f). Without tamoxifen treatment, we did not detect any tdTomato^+^ cell in the blood, spleen, or bone marrow, indicating no leakiness of *CD11b-CrexER*; while tamoxifen treatment resulted in specific labeling of CD11b^+^ cells (Supplementary Fig. S1g, h), indicating specificity. Having successfully generated the *Myh11-Dre* and *CD11b-CrexER* mouse lines, we crossed them to develop a sequential intersectional genetic strategy in which *Myh11-Dre*-mediated Dre-rox recombination first removed ER from the CrexER cassette, thus switching *CD11b-CrexER* into the *CD11b-Cre* genotype in smMHC^+^ SMCs (Step 1, Fig. 1a). CD11b-expressing macrophages originating from smMHC^+^ SMCs were, therefore, genetically labeled by the *R26-tdT* reporter, and their subsequent cell fate in the atherosclerotic plaques could be traced and analyzed using tdTomato (Step 2, Fig. 1a). By crossing with *LDLR*^−/−^, we generated the *Myh11-Dre;CD11b-CrexER;R26-tdT;LDLR*^−/−^ line. First, we analyzed the tdTomato signaling of *Myh11-Dre;CD11b-CrexER;R26-tdT;LDLR*^−/−^ mice line in homeostasis. We did not detect any tdTomato^+^ cells in the aorta, blood, spleen, or bone marrow (Fig. 1b). We then fed mice on a high-fat diet (HFD) or normal diet (ND) starting at 8 weeks of age, and analyzed tissues at 16 or 32 weeks of age (Fig. 1c). HFD treatment led to macrophage accumulation in atherosclerotic plaques at 16 weeks of age, and a few tdTomato^+^ cells were detected among them (Fig. 1d). We stained tissues with antibodies for macrophage markers, CD45, CD11b, F4/80, and CD68; or with smooth muscle cell marker aSMA. The immunostaining results revealed that all tdTomato^+^ cells expressed these macrophage markers, but not aSMA (Supplementary Fig. S2a, b). Furthermore, we confirmed this result by multicolor cell flow cytometry. The tdTomato^+^ cells at this early stage were mostly CD45^+^CD11b^+^F4/80^+^ macrophage-like cells (Supplementary Fig. S2c, d). In addition, the tdTomato^+^ cells constitute only ~0.146% of total macrophages (Supplementary Fig. S2c, e), which was lower than other models as previously reported^3^. This maybe caused by the different time points analyzed and markers used here. Of note, we did not detect any tdTomato^+^ cells in the blood, spleen, or bone marrow (Supplementary Fig. S2f).

**Fig. 1:**
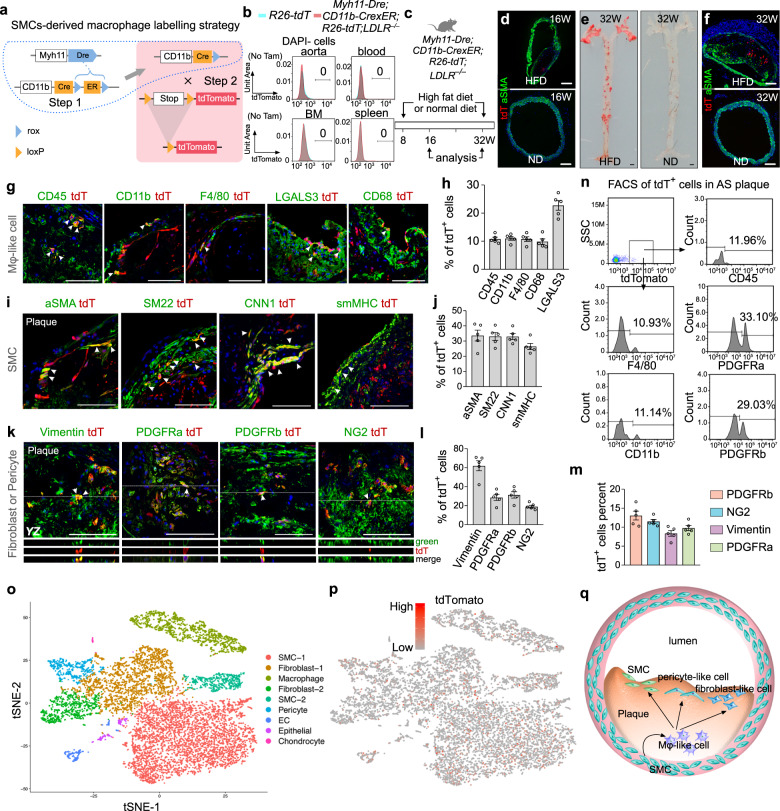
Fate mapping of SMC-derived macrophage-like cells in advanced atherosclerotic plaques of 32-week-old mice. **a** Schematic diagram showing the dual recombinase-mediated sequential intersectional genetic strategy. Step 1: Dre-rox recombination switches *CD11b-CrexER* to the *CD11b-Cre* genotype in smMHC^+^ cells; Step 2: when CD11b is activated, Cre-loxP recombination genetically labels CD11b-expressing cells. **b** Flow cytometric analysis of tdTomato^+^ cells in the blood, spleen, and bone marrow of *Myh11-Dre;CD11b-CrexER;R26-tdT;LDLR*^−/−^ mice. **c** Schematic diagram showing the experimental strategy. Mice were treated with a high-fat diet (HFD) or normal laboratory diet. **d** Immunostaining for tdT and aSMA on aortic sections from 16-week-old mice treated with HFD or normal diet. **e** Whole-mount images of aortas after Sudan IV staining. **f** Immunostaining for tdTomato (tdT) and aSMA in aortic sections from mice treated with HFD or normal diet. **g** Immunostaining for tdT and CD45, CD11b, F4/80, LGALS3, or CD68 on plaque sections. Arrowheads, tdT^+^ macrophages. **h** Quantification of % of tdT^+^ cells expressing different macrophage markers in the plaques. Data are expressed as means ± SEM; *n* = 5. **i** Immunostaining for tdT and SMC markers. Arrowheads, tdT^+^ SMCs. **j** Quantification of the % of tdT^+^ cells expressing different SMC markers in the plaques. Data are expressed as means ± SEM; *n* = 5. **k** Immunostaining for tdT and fibroblast or pericyte markers. Arrowheads, tdT^+^ fibroblasts or pericytes. **l** Quantification of the % of tdT^+^ cells expressing fibroblast or pericyte markers in the plaques. Data are expressed as means ± SEM; *n* = 5. **m** Quantification of the % of fibroblasts or pericytes expressing tdT in the plaques. Data are expressed as means ± SEM; *n* = 5. **n** Flow cytometric analysis of different lineage markers expressed by tdT^+^ cells isolated from the plaques. **o** tSNE analysis of scRNA-seq data of 10,618 plaque cells from *Myh11-Dre;CD11b-CrexER;R26-tdT;LDLR*^*−/−*^ mice fed with 24 weeks HFD. **p** Feature plot of tdTomato in different clusters. 1946 tdT^+^ cells are presented in the clusters. **q** Cartoon image showing a subset of SMC-derived macrophage-like cells adopting a transient cell fate and contributing to SMCs, pericyte-like cells, and fibroblast-like cells in the fibrous cap and atherosclerotic plaques. Scale bars: black, 1 mm; white, 100 µm. Each image is a representative of five individual biological samples.

While the tdTomato^+^ cells were exclusively macrophages at 16 weeks old, we next asked whether SMCs-derived macrophages are terminally differentiated or being in an intermittent state in which they could further adopt other cell fate(s) in the advanced atherosclerotic plaques at 32 weeks old. Treatment of HFD but not ND led to the formation of advanced atherosclerotic lesions in the aorta at 32 weeks old (Fig. 1e) that consisted of readily detectable tdTomato^+^ cells (Fig. 1f). To determine their cell fate, we performed immunostaining for tdTomato and multiple cell lineage markers on frozen sections of the advanced plaques. We found that only a subset of tdTomato^+^ cells (~10%) still maintained expression of macrophage cell markers such as CD45, CD11b, F4/80, or CD68 (Fig. 1g, h), indicating that a substantial number of tdTomato^+^ cells might no longer maintain the macrophage cell fate. A large proportion of tdTomato^+^ cells (~25%–35%) in the fibrosis cap of the atherosclerotic plaques expressed SMC markers such as aSMA, SM22, CNN1, and smMHC (Fig. 1i, j), indicating that these macrophages may revert to SMC fate during the progression of atherosclerosis. Furthermore, immunostaining for tdTomato and fibroblast or pericyte markers showed that a subset of tdTomato^+^ cells expressed Vimentin (~60%), PDGFRa (~25%), PDGFRb (~30%), or NG2 (~18%) in the atherosclerotic plaques (Fig. 1k, l), indicating that SMC-derived macrophages contributed to fibroblasts and pericyte-like cells in the advanced atherosclerotic lesions. The macrophage-like cells expressing fibroblasts or pericytes markers occupied ~10% among total fibroblasts and pericyte-like cells in the plaques (Fig. 1m). It is possible that some other fibroblasts and pericytes could be derived from other sources, not SMC-derived macrophage-like cells. Flow cytometric analysis of tdTomato^+^ cells purified from the atherosclerotic plaques revealed that ~10% tdTomato^+^ cells expressed macrophage markers, and ~30% tdTomato^+^ cells expressed PDGFRa or PDGFRb (Fig. 1n). To further validate the cell lineages of tdTomato^+^ cells, we performed scRNA-seq of 10,618 advanced plaque cells (Fig. 1o, Supplementary Fig. S3). 1946 tdTomato^+^ cells were detected in the cell clusters that have enriched signature for macrophages, smooth muscle cells, fibroblasts, and pericytes (Fig. 1p), suggesting SMC-derived macrophage-like cells contribute to cells expressing these multiple cell lineage markers. In three different sets of technical controls, *Myh11-Dre;CD11b-CrexER;R26-tdT;LDLR*^*–/+*^, *Myh11-Dre;R26-tdT;LDLR*^−/−^, and *CD11b-CrexER;R26-tdT;LDLR*^−/−^ mice, we did not detect any tdTomato^+^ cell in the valves or vessel walls of the aortas (Supplementary Fig. S2g), demonstrating that SMC-derived macrophages could only be detected by a dual genetic tracing system during the progression of atherosclerosis.

Recent study using dual recombination system, *Myh11-DreER;Lgals3-RSR-Cre*, revealed the cell fate transition of SMC-derived LGALS3^+^ cells^6^. They also observed SMC-derived macrophages expressing CD11b and F4/80. In our study, SMC-derived macrophage-like cells (tdTomato^+^ cells) express CD45, CD11b, F4/80, and CD68 (Supplementary Fig. S2a-d) in the early stage lesion. We also performed scRNA-seq analysis of cells isolated from atherosclerotic plaque and found that CD11b was enriched in the macrophage population (Supplementary Fig. S3e), suggesting CD11b reflect macrophage or macrophage-like status of SMCs during atherosclerosis. In the study by Wirka et al.^7^, they reported that SMCs transformed into fibroblast-like cells rather than into a classical macrophage phenotype. Our lineage tracing added new information that the contribution of SMCs to fibroblast-like cells has transiently expressed macrophage markers during atherosclerosis. Recent study reported that SMCs also transitioned to an intermediate cell state during atherosclerosis, as they differentiate into macrophage-like and fibrochondrocyte-like cells, as well as return toward to SMC phenotype^8^. Our study provided direct genetic evidence of conversion of the SMC-derived macrophage-like cells to cells expressing other cell lineage markers in plaques. Besides, we speculate that our observation could be also conserved in ApoE^−/−^ model, as recent studies reported the cell fate transition of SMC-derived LGALS3^+^ cells in ApoE^−/−^ mice^6^ and the SMCs-derived intermediate cell state in both ApoE^−/−^ and LDLR^−/−^ mice^8^.

In this study, We used *Myh11-Dre;CD11b-CrexER* to trace SMC-derived macrophage-like cells in atherosclerosis. Our findings suggested that while a subset of SMC-derived macrophage-like cells continued to be macrophage-like cells in the advanced plaques, the majority of them did not continue to express macrophage markers at late stage. Instead, some of these SMC-derived macrophage-like cells contribute to cells expressing fibroblast or pericyte markers in the atherosclerotic plaques (Fig. 1q). This study also demonstrated that macrophage-like cells derived from SMCs in the plaques could exhibit a transient cell fate with the potential to express markers of multiple cell lineages during the progression of atherosclerosis. Our data revealed the distinct cell plasticity of SMCs-derived macrophage-like cells, which differed from those recruited from monocytes that are known to be terminally differentiated. Usually, macrophages were known as promoting atherosclerosis progress, expanding lesion size, and contributing to plaque rupture. The role of smooth muscle cell-derived macrophage-like cells in atherosclerosis was under controversial. Here, we find that SMC-derived macrophage-like cells could revert to smooth muscle cells in fibrous cap of advanced plaques, suggesting that these cells may play a protective role by stabilizing atherosclerotic plaques and exert a repair mechanism in atherosclerosis. Further elucidation of the origin and plasticity of multiple cell lineages in the plaques, as well as their potential functions in the future may aid in the development of potential therapeutic targets assisting treatment of atherosclerosis. The mechanism controlling SMC-derived macrophage-like cell fate modulation and how this fate transition impacts on the stability of plaque merits further investigation in future.

## Supplementary information

Supplementary Information

## Data Availability

The data that support the findings of this study and research materials, as well as experimental procedures and protocols, are available from the corresponding author upon request.
