# Supplementary material for: Smooth muscle-derived macrophage-like cells contribute to multiple cell lineages in the atherosclerotic plaque
Source: Cell Discov. 2021 Nov 23;7:111. doi: 10.1038/s41421-021-00328-4 (PMC8608914; doi:10.1038/s41421-021-00328-4)
Supplement: Supplementary file 1 — Supplementary Information [file 41421_2021_328_MOESM1_ESM.pdf]

Supplementary Materials for

Smooth Muscle-derived Macrophage-like Cells Contribute to Multiple Cell Lineages in the Atherosclerotic Plaque

Yi Li[1,2], Huan Zhu[2], Qianyu Zhang[3], Ximeng Han[2], Zhenqian Zhang[2], Linghong Shen[1], Lixin Wang[4], Kathy O. Lui[5], Ben He[1], Bin Zhou[2,3,6]

[1]Department of Cardiology, Shanghai Chest Hospital, Shanghai Jiaotong University, Shanghai, China.

[2]State Key Laboratory of Cell Biology, Shanghai Institute of Biochemistry and Cell Biology, Center for Excellence in Molecular Cell Science, Chinese Academy of Sciences; University of Chinese Academy of Sciences, Shanghai 200031, China.

[3]School of Life Science and Technology, ShanghaiTech University, 100 Haike Road, Shanghai 201210, China.

[4]Department of Cardiac Surgery, Zhongshan Hospital, Fudan University, Shanghai, China.

[5]Department of Chemical Pathology; and Li Ka Shing Institute of Health Sciences, The Chinese University of Hong Kong, Prince of Wales Hospital, Shatin, Hong Kong SAR, 999077, China

[6]School of Life Science, Hangzhou Institute for Advanced Study, University of Chinese Academy of Sciences, Hangzhou 310024, China.

Contents:

Methods and Materials
Supplementary Fig. S1-S3

Methods and Materials

The data, analytic methods, and study materials that support the findings of this study are available from the corresponding author upon reasonable request.

Experimental mice

All mouse experiments were done with the approval of the Institutional Animal Care and Use Committee (IACUC) at the Institutes for Biochemistry and Cell Biology, Shanghai institutes for Biological Sciences, Chinese Academy of Sciences. Both male and female mice were analyzed in this study. The mouse background is a mixture of C57BL/6J and ICR. The *Myh11-Dre* mouse line was generated by knock-in of Dre cDNA through homologous recombination using CRISPR/Cas9 strategy and direct editing in mouse zygotes. We inserted Dre-WPRE-polyA sequence into the Myh11 gene locus by replacing ATG of endogenous gene. 4 *Myh11-Dre* knock-in mice were got from 21 zygotes. The plasmid containing Dre was originally obtained from Konstantinos Anastassiadis and Francis Stewart at Dresden, Germany. The *CD11b-CrexER* mouse line was generated by knock in of Cre-rox-ER-rox cDNA through homologous recombination using CRISPR/Cas9 strategy and direct editing in mouse zygotes. After characterizing, we got 1 CD11b-CrexER knock-in mice from 122 zygotes. We generated Cre-rox-ER-rox-WPRE-polyA cassette and insert it into CD11b gene locus by replacing the endogenous ATG. Shanghai Model Organisms Center, Inc. (SMOC) generated the *Myh11-Dre* and *CD11b-CrexER* mouse line. *LDLR-/-* mice were reported previously[1]. The mice were fed on high fat diets (Dyets, ASHF3, 1.25% cholesterol) for 16-32 weeks for induction of atherosclerosis and normal laboratory diet (Jiangsu Xietong Pharmaceutical Bioengineering, 1010085) as control.

Genomic PCR

Mouse genomic DNA was extracted from tails. Tissues were incubated in 250μL lysis buffer containing 100 μg/ml proteinase K overnight at 55°C. After vortex for 10s, the buffer was centrifuged at 15000rpm for 5min to obtain supernatant with genomic DNA. Then adding equal volume isopropanol to precipitate DNA and washed in 70% ethanol. These steps need centrifugation at 15000rpm for 3min to obtain precipitate with genomic DNA. All DNA was genotyped with specific primers that distinguished the knock-in allele from the wild-type allele. For *R26-tdTomato* line, primers 5'-AAGGGAGCTGCAGTGGAGTA-3' and 5'-CCGAAAATCTGTGGGAAGTC-3' were used to detect the wild type allele. Primers 5'-GGCATTAAAGCAGCGTATCC-3', and 5'- CTGTTCCTGTACGGCATG-3' were used to detect *R26-tdTomato* inserted allele. For *CD11b-CrexER* line, primers 5'-TCCTCAAAGTGTCTGTGGTGCCTG-3' and 5'- TCTTCAGGTTCTGCGGGAAAC-3' were used to detect inserted allele. Primers 5'-TCCTCAAAGTGTCTGTGGTGCCTG-3' and 5'-TATTCCTCCTGCCTACCTCTGCTC-3' were used to detect wild type allele. For *Myh11-Dre* line, primers 5'-TGACCCCATCTCTTCACTCCACAG-3' and 5'-

CACCCAGGCATCGCTAAAAATC-3’ were used to detect *Myh11-Dre* inserted allele. Primers 5’-TGACCCCATCTCTTCACTCCACAG-3’ and 5’-CCATTTTCCACCAACTCCACG-3’ were used to detect wild type allele. For *CAG-Dre* line, primers 5’-ACTCCTTGCCGATGTTCCTCAG-3’ and 5’-TTGTCCCAAATCTGGCGGAG-3’ were used to detect inserted allele. For *LDLR-/-* line, primers 5’-AAGACGTGCTCCCAGGAT-3’ and 5’-ATCGCCTTCTTGACGAGTTC-3’ were used to detect knock out allele. Primers 5’-AAGACGTGCTCCCAGGAT-3’ and 5’-CGTGCTCCTCATCTGACTTGT-3’ were used to detect wild type allele. For *R26-rox-tdTomato* line, primers 5’-ACGGGTGTTGGGTCGTTTGTTC-3’ and 5’-TTCTTGTAATCGGGGATGTCGGCG-3’ were used to detect inserted allele. Primers 5’-AAGGGAGCTGCAGTGGAGTA-3’ and 5’-CCGAAAATCTGTGGGAAGTC-3’ were used to detect wild type allele. For *R26-rox-ZsGreen* line, primers 5’-CAAGGAGATGACCATGAAGTACCG-3’ and 5’-GGGCAGGAGTTCTTGAAGTAGTCG-3’ were used to detect inserted allele. The programs of PCR used 60°C as the annealing temperature and 35 cycles for amplification of genomic DNA.

Immunofluorescent staining

Tissues were collected in PBS, and then fixed in 4% PFA for 50-60 minutes in 4°C. After three times of washing in PBS for 15 minutes, tissues were dehydrated in 30% sucrose (dissolved in PBS) for 6-8 hours and embedded in OCT (Sakura). Next, cryosections of 9-10μm in thickness were collected. After air dry for 1-1.5 hour at room temperature, sections were incubated with 30% H_2O_2 for 15 minutes. After two times of washing in PBS for 15 min at room temperature, slides were blocked in blocking buffer (5% donkey serum, 0.1% Triton X-100 in PBS) for 40 min at room temperature, followed by incubation with primary antibodies (SMA-FITC, Sigma, F3777; CD45, eBioscience, 17-0451-82; CD11b, Thermo Fisher Scientific, 14-0112-82; tdTomato, Rockland, 600-401-379; F4/80 Abcam, ab6640; CD68, Bio-Rad, MCA1957; CNN1, Abcam, ab46794; smMHC, Abcam, ab53219; Vimentin, Cell signaling, 5741; PDGFRa, R&D, AF1062; PDGFRb, eBioscience, 14-1402-82) at 4°C overnight. On the next day, slides were washed two times in PBS for 15 min and then incubated with secondary antibodies (Immpress anti-rat, Vector Lab, MP-744-15; donkey anti-rabbit 555, Invitrogen, A31572; Immpress anti-rabbit, Vector Lab, MP-7401) for 40 min at room temperature in dark. For PDGFRa, PDGFRb, NG2 and Vimentin staining, we used HRP-conjugated antibodies (Vector Lab) with tyramide signal amplification kit (PerkinElmer) to develop the signal for 5 minutes in dark at room temperature. After three times of washing in PBS for 15 min at room temperature, tissue sections were mounted with mounting medium containing the nuclear stain DAPI (Vector Lab). Images were acquired on Zeiss LSM710. The obtained images were analyzed by ImageJ software (NIH). We collected over 30 tissue sections from each sample of 5 individual mice in each group, and stained tissue sections with tdTomato and cell lineage markers. For quantification of the percentage of tdTomato$^+$ cells expressing different cell lineage markers, the number of Marker$^+$tdTomato$^+$ cells is set as

numerator while the number of tdTomato$^+$ cells is set as denominator. This result as ratio is then transformed into percentage. For quantification of the percentage of cells (e.g. fibroblasts, pericytes et al.) expressing tdTomato, the number of Marker$^+$tdTomato$^+$ cells is set as numerator while the number of Marker$^+$ cells is set as denominator. This result as ratio is then transformed into percentage.

Aortic cell isolation and flow cytometry

The aortas were harvested from the mice perfused with PBS, and then finely minced and transferred to the digestion mix (HBSS, 2U/ml Liberase (5401127001, Sigma) and 2 U/ml elastase (LS002279, Worthington)). Tissues were incubated with the digestion mix at 37°C for 30-40 minutes. The cell suspension was filtered through 70 μm cell strainer and then pelleted by centrifugation at 400g for 5min. The precipitated cells were re-suspended with PBS and then stained with violet dye at 4°C for 30 minutes. After washing cells with 500ml PBS, cells were centrifuged for 2000rpm for 3 minutes and the supernatant was discarded. Cells were separately stained with CD45-PE-Cy7 (eBioscience, 25-0451-82), CD11b-PerCP-Cy5.5 (eBioscience, 45-0112-82), F4/80-FITC (eBioscience, 11-4801-82), CD45-APC (eBioscience, 17-0451-82), CD11b-APC (eBioscience, 17-0112-81), F4/80-APC (eBioscience, 17-4801-82), PDGFRa-APC (BD pharmingen, 562777) and PDGFRb-APC (eBioscience, 17-1402) for 30 minutes at 4°C. Finally, the cells were re-suspended in PBS and analyzed with CytoFlex LX (Beckman). The flow cytometric data was analyzed by the Flowjo software.

scRNA-seq

The plaques cells were isolated as above method. DAPI- live cells were loaded in 10x Chromium controller the generate GEMs, which were further processed into single cell 5' gene expression libraries using Chromium Single Cell 5' Library Kit (v1.1) . Sequencing was performed on Illumina Hiseq X Ten PE150 platform. Fastq files were processed by CellRanger (v.4.0.0) pipeline. tSNE dimplot, dotplot and featureplot were analyzed by R package Seurat (v.4.0.1) and dplyr (v.1.0.5) with customized parameters.

Statistical analysis

All data were representative of 5 individuals, as indicated in each figure legend, and presented as mean values ± SEM.

Reference

1 Ishibashi, S. *et al.* Hypercholesterolemia in low density lipoprotein receptor knockout mice and its reversal by adenovirus-mediated gene delivery. *J Clin Invest* **92**, 883-893 (1993).

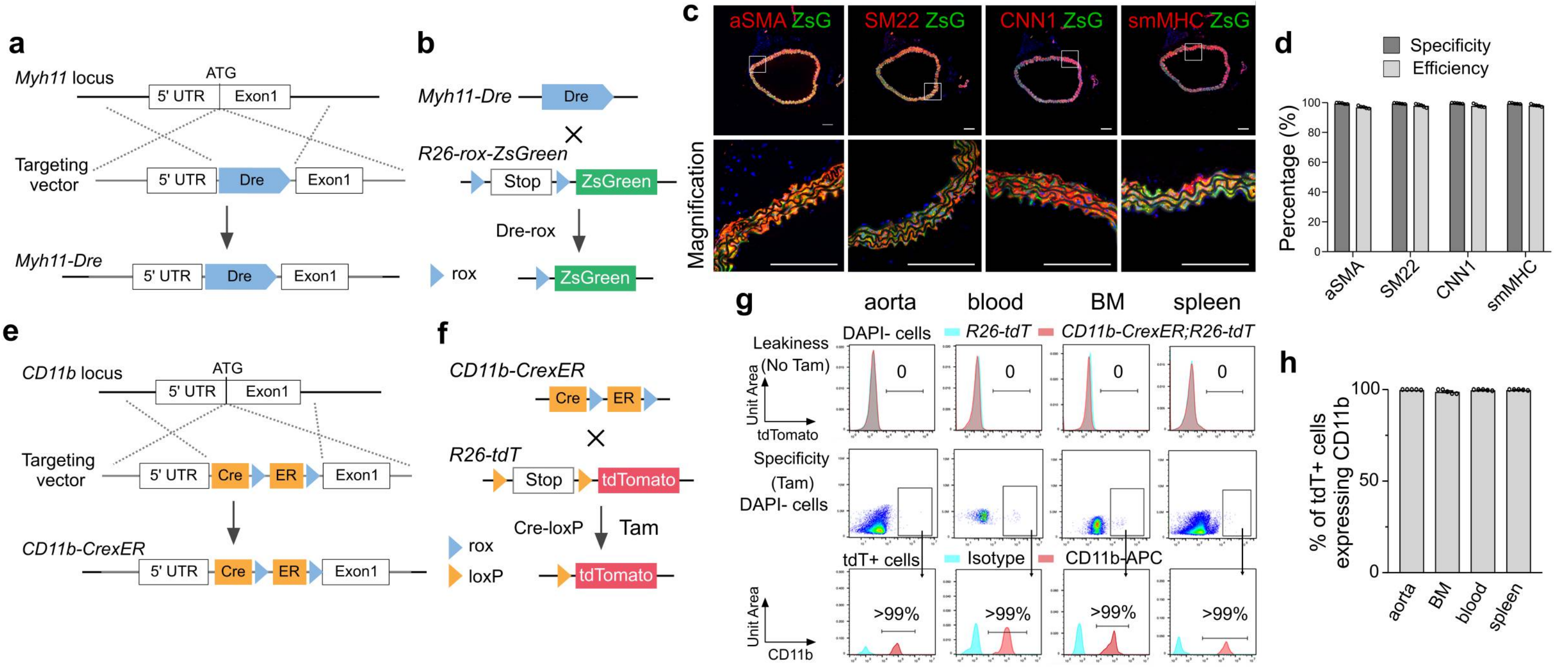

Supplementary Fig. S1 Generation and characterise of *Myh11-Dre* and *CD11b-CrexER* mice. (a) Schematic diagram showing the strategy for the generation of *Myh11-Dre* knock-in mice by homologous recombination using CRISPR/Cas9. (b) Schematic figure showing the labelling of SMCs by crossing with the rox reporter. (c) Immunostaining on aortic sections derived from *Myh11-Dre;R26-rox-ZsGreen* mice. (d) Quantification of the percentage (%) of ZsGreen+ cells expressing SMC markers (specificity) and the % of SMC marker+ cells expressing ZsGreen (efficiency). Data are expressed as mean ± SEM; n = 5. (e) Schematic diagram showing the strategy for the generation of *CD11b-CrexER* knock-in mice by homologous recombination using CRISPR/Cas9. (f) Schematic figure showing the crossing of *CD11b-CrexER* with the loxP reporter. (g) Flow cytometric analysis of tdTomato+ cells in the blood, spleen, and bone marrow of *CD11b-CrexER;R26-tdT* mice without tamoxifen treatment (No Tam, upper panel, gated from DAPI- cells) and the percentage of tdTomato+ cells expressing CD11b in mice 2 days after Tam treatment (lower panel, gated from tdTomato+ cells (middle panel)). (h) Quantification of the specificity of *CD11b-CrexER*-labeled cells 2 days after Tam treatment. Scale bars, 100 μm. Each image is a representative of 5 individual biological samples.

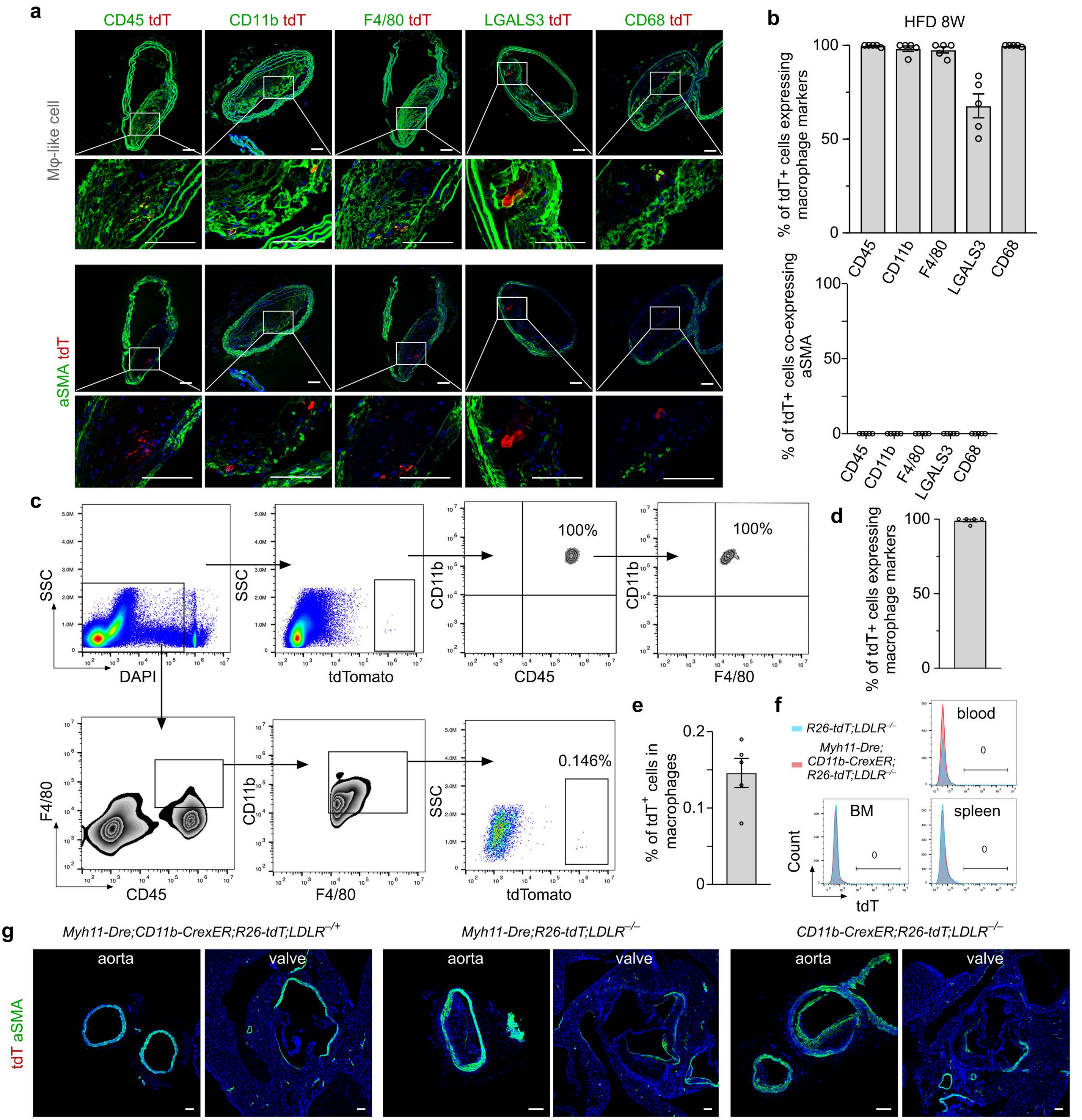

Supplementary Fig. S2 tdTomato labeled cells in early plaques of 16 weeks old mice are macrophage-like cells. (a) Staining of CD45, CD11b, F4/80, LGALS3, CD68, and aSMA with tdTomato in plaques. (b) Quantification of tdTomato+ cells expressing CD45, CD11b, F4/80, LGALS3, and CD68 (upper panel). Quantification of tdTomato+ cells co-expression of CD45, CD11b, F4/80, LGALS3, and CD68 with aSMA (lower panel). (c) Cell flow cytometry of plaque cells from *Myh11-Dre;CD11b-CrexER;R26-tdT;LDLR-/-* mice treated with 8weeks HFD. (d) Quantification of tdTomato+ cells expressing macrophage markers, CD45, CD11b and F4/80. (e) Quantification the percentage of tdTomato+ cells in macrophages. (f) Cell flow cytometry of blood, bone marrow, spleen cells from *Myh11-Dre;CD11b-CrexER;R26-tdT;LDLR-/-* mice treated with 8weeks HFD. Scale bars, 100 μm. Each image is a representative of 5 individual biological samples. Quantification data are expressed as mean ± SEM. (g) Immunostaining for tdT and aSMA in three different mouse lines fed on HFD.

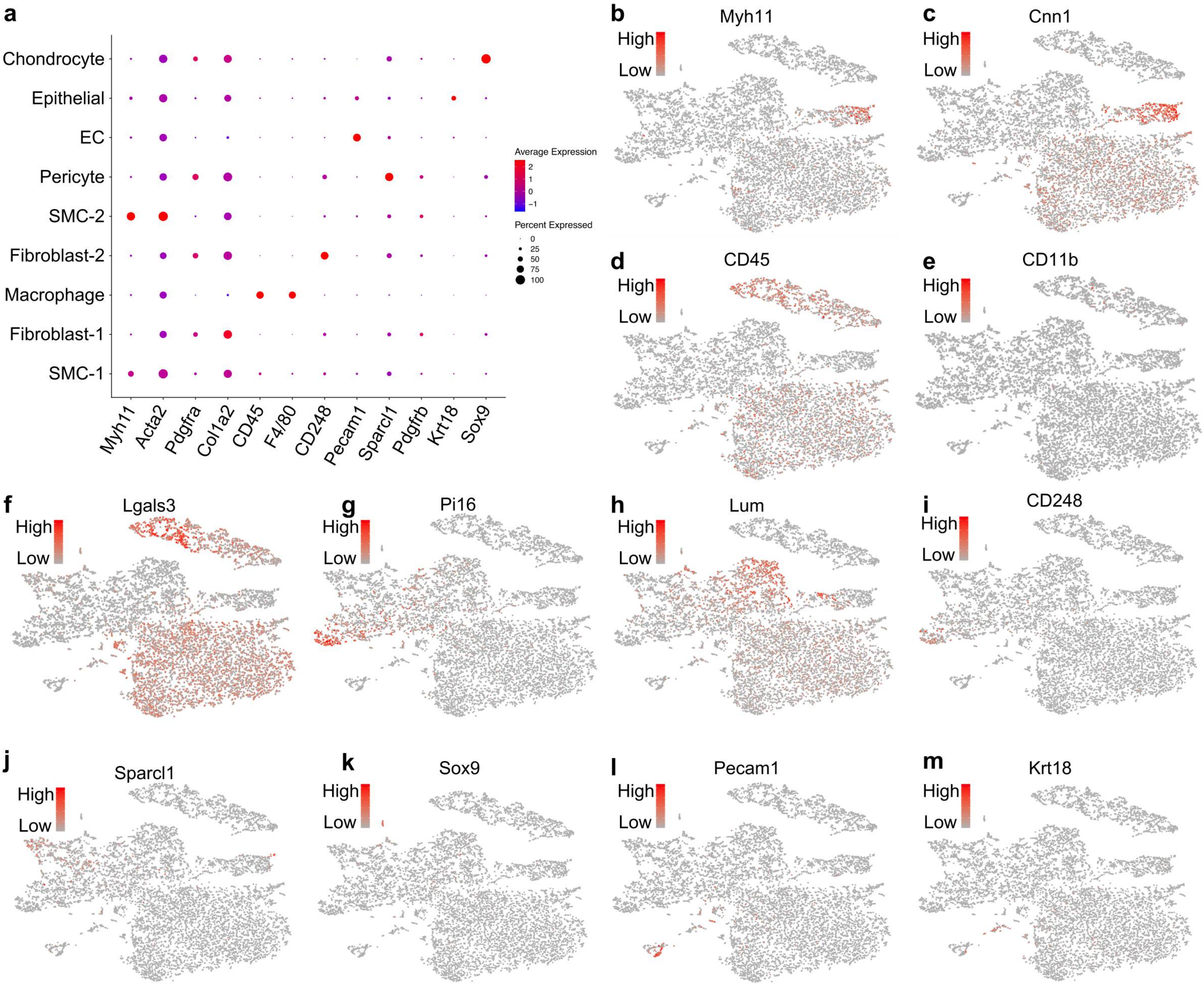

Supplementary Fig. S3 scRNA-seq analysis of plaque cells from mice fed with 24 weeks HFD. (a) The representative genes define each cell clusters. (b, c) Feature plots of smooth muscle cell markers, Myh11 and Cnn1. (d-f) Feature plots of macrophage markers, CD45, CD11b and Lgals3. (g-i) Feature plots of fibroblast markers, Pi16, Lum and CD248. (j) Feature plots of pericyte marker, Sparcl1. (k) Feature plots of chondrocyte marker, sox9. (l) Feature plots of endothelial cell marker, Pecam1. (m) Feature plots of epithelial marker, Krt18. SMC-1 also expressed some fibroblast markers (g, h), suggested this cluster cells were mostly likely modulated SMCs. Myh11 was uniquely expressed in smooth muscle cells (b). CD11b was mostly expressed by macrophages (e).
